# Detecting individual ancestry in the human genome

**DOI:** 10.1186/s13323-015-0019-x

**Published:** 2015-05-01

**Authors:** Andreas Wollstein, Oscar Lao

**Affiliations:** Department of Forensic Molecular Biology, Erasmus MC University Medical Center Rotterdam, 3000 CA Rotterdam, The Netherlands; Section of Evolutionary Biology, Department of Biology II, University of Munich, 82152 Planegg-Martinsried, Germany; Current address: Centro Nacional de Análisis Genómico, Baldiri Reixac, 4, Barcleona Science Park - Tower I, 08028 Barcelona, Spain

**Keywords:** Population substructure, Human genetic variability, SNPs, Global ancestry, Individual ancestry, ADMIXTURE, fastSTRUCTURE, MDS, PCA, sNMF

## Abstract

Detecting and quantifying the population substructure present in a sample of individuals are of main interest in the fields of genetic epidemiology, population genetics, and forensics among others. To date, several algorithms have been proposed for estimating the amount of genetic ancestry within an individual. In the present review, we introduce the most widely used methods in population genetics for detecting individual genetic ancestry. We further show, by means of simulations, the performance of popular algorithms for detecting individual ancestry in various controlled demographic scenarios. Finally, we provide some hints on how to interpret the results from these algorithms.

## Review

### Introduction

The genetic variability among the human species is known to be relatively low compared to other primate species [1]. There are paradoxically more genetic differences between Western and Eastern chimpanzee individuals sampled in the African continent [2] than in any genome of two human individuals sampled in different continents [3]. Human genetic diversity also tends to be positively correlated with the geographic distance between the sampled individuals [4-6], which is mainly a result from isolation by distance [7]. Studies using classical partition of the human genetic variance based on analysis of molecular variance (AMOVA [8]), and its generalization GAMOVA [9], have consistently shown that a small proportion (approximately 10% to 15%) of the total genetic variability is explained by continent of origin, whereas the majority (approximately 80%) is explained by within-individual variation. The remaining approximately 5% of the genetic variation is explained by the populations [10]. Interpreting these results in terms of human population substructure and individual prediction to a population cluster is still controversial [11]. Some argue that humans should be considered as one genetically homogeneous group [12]; others suggest that, although small, the geographic dependence of human genetic diversity (at least) supports the existence of continental groups [11,13].

Inferring population substructure in the human genome is cumbersome and is the main goal for the large number of genetic ancestry algorithms and approaches that have been proposed in the last decade. A basic assumption is that any current individual genome or population is a mixture of ancestries from past populations [14]. Therefore, genetic ancestry is defined at different scales of complexity: at populations, at individuals within a population, and at a locus within an individual. In the present review, we focus on current methods for inferring genetic ancestry in the genome of an individual. We analyze the performance of some of the most commonly used programs through simulated data and show the range of parameters in which each program provides reliable results in those settings.

### Methods for identifying individual ancestry

Methods for estimating ancestry have traditionally focused on populations; their main interests are to establish the relationship among populations and to quantify the admixture proportions in the admixed populations [15,16]. Admixture proportions are computed from the amount of loci that can be traced back to a certain ancestral population. Population methods are the oldest in literature [17] and are a large number of available applications [18-21]. However, it has been suggested that there could be hidden population substructure among the individuals from an assumed population [22]. The main goal of global individual ancestry methods is to describe the relationship between individuals in terms of genetic ancestry. This can either mean the identification of the *a priori* unknown ancestry components, the quantification of the proportions of these components, or the identification of the assumed population of an individual. Individual ancestry methods can be classified depending on the assumptions of the method, the scope of the algorithms (that is, the whole genome is assigned to one ancestry *versus* the whole genome is a mixture of ancestries), and the use of prior ancestry information, among others (see Table 1). From a technical point of view, there is large variation in the speed and computational requirements of the different methods [16,23]. Speed depends on the computational complexity of each method which, for example, is O(*n m K*^*2*^) for ADMIXTURE [24] and O(*n m K*) for sNMF [25], as well as the possibility to apply divide-and-conquer computational approaches such as multithreading (for example, in ADMIXTURE and sNMF). However, multithreading can only provide a linear time factor gain, which in the case of higher polynomial complexities does not have a strong computational impact.

**Table 1:** **Commonly applied algorithms to SNP data for quantifying individual population substructure in humans**

**Type**	**Method**	**Name of package**	**Web address**	**Reference**
Model-free	Principal component analysis	EIGENSOFT^a^	http://genetics.med.harvard.edu/reich/Reich_Lab/Software.html	[70]
	Principal components and Moran’s*I*	adegenet (R software)	http://adegenet.r-forge.r-project.org/	[71]
	Multidimensional scaling	PLINK^a^	http://pngu.mgh.harvard.edu/~purcell/plink/	[28]
	Principal coordinates	PCO-MC	http://lamar.colostate.edu/~reevesp/PCOMC/PCOMC.html	[72]
	Spectral graph theory	GemTools	http://wpicr.wpic.pitt.edu/WPICCompGen/GemTools/GemTools.htm	[43]
	Spectral graph theory	SpectralGem	http://wpicr.wpic.pitt.edu/WPICCompGen/Spectral-GEM/GEM+.htm	[56]
	Laplacian eigenfunction	LAPSTRUCT	http://galton.uchicago.edu/~junzhang/LAPSTRUCT.html	[57]
	Genetic algorithm coupled to AMOVA	GAGA	http://www.erasmusmc.nl/fmb/resources/GAGA/	[73]
Model-based	Log-likelihood HWE	ADMIXTURE	https://www.genetics.ucla.edu/software/admixture/	[24]
	Log-likelihood HWE	FRAPPE	http://med.stanford.edu/tanglab/software/frappe.html	[31]
	Bayesian HWE	STRUCTURE	http://pritchardlab.stanford.edu/structure.html	[22]
	Bayesian HWE	fastSTRUCTURE	http://pritchardlab.stanford.edu/structure.html	[59]
	Nonnegative matrix factorization	sNMF	http://membres-timc.imag.fr/Eric.Frichot/snmf/index.htm	[25]
	Bayesian	BAPS	http://www.helsinki.fi/bsg/software/	[74]
	Chromopainting and Bayesian classifier	fineSTRUCTURE	http://www.paintmychromosomes.com	[60]
	Log-likelihood genotypic/haplotypic gradients	LOCO-LD	http://loco.icsi.berkeley.edu/loco/	[37]
	Log-likelihood allelic gradients	SPA	http://genetics.cs.ucla.edu/spa/	[36]
	ADMIXTURE and linear regression	GPS	http://chcb.saban-chla.usc.edu/gps/	[39]
	Bayesian clustering with spatial information	TESS	http://membres-timc.imag.fr/Olivier.Francois/tess.html	[38]

^a^We provide one of the possible implementations present in the literature.

Depending on which methodological approach is used, global individual ancestry methods have been divided by Alexander *et al.* [24] into algorithmic and model-based methods [24]. We use this classification through the manuscript with some modifications. By definition, all the algorithms are ‘algorithmic’. Therefore, we will use the term ‘model-free’ for referring to the ancestry methods classified by Alexander *et al.* [24] as algorithmic, and point out that the use of ‘model’ refers here to a population-based statistical model, as further described. Nevertheless, we acknowledge that some of the newest proposed methods can also be considered as hybrids of the two classifications or even can be barely assigned to any of them. Model-free methods are based on the use of multivariate techniques [26] such as Principal component analysis (PCA; [27]) or Multidimensional scaling (MDS [28,29]). For a given measured divergence between any pair of sampled individuals, the basic idea behind all these techniques is to represent the genetic relationships by a new set of orthogonal variables that are ordered by the decreasing amount of explained variation. Both methods can be considered as equivalent if Euclidean distances are used [29]. Visualization of these relationships becomes very meaningful if only the variables with the highest amount of explained variation are considered. Because multivariate methods are exploratory, they do not make any assumption about the underlying genetic model of the data [26]. Nevertheless, in some idealized cases, the proposed coordinates in some of these methods can be interpreted in demographic terms (for example, PCA [30]). In contrast, model-based methods estimate ancestry coefficients as the parameters of a statistical model. This model takes into account basic demographic assumptions, such as the presence of the Hardy-Weinberg equilibrium (HWE; [22]) in the allelic frequencies of the *K* ‘ancestral’ populations that produced the currently observed data [22,24]. For example, in the original definition of individual ancestry provided by STRUCTURE [22], the genotype *g* counted as the number of alleles {0,1,2} in a diploid organism at locus *j* of individual *i* is modeled as a mixture of the *q* fractions of the *K* ancestral populations at the allelic frequencies *f*. The log-likelihood under the assumption of HWE for all the individuals *i* and loci *j* is then computed using the Alexander *et al.* [24] notation as:

$$ L\left(Q,F\right)={\displaystyle \sum_i{\displaystyle \sum_j\left({g}_{ij} \ln \left({\displaystyle \sum_k}{q}_{ik}{f}_{kj}\right)+\left(2-{g}_{ij}\right) \ln \left({\displaystyle \sum_k}{q}_{ik}\left(1-{f}_{kj}\right)\right)\right)}} $$

Popular methods for estimating the allelic frequencies *f* in the ancestral populations for all the loci and the ancestry *q* proportions in each individual include Bayesian (for example STRUCTURE [22]) and maximum likelihood approaches (for example, FRAPPE [31] and ADMIXTURE [24]).

Recently, new types of global ancestry methods have been proposed. These methods take advantage of the spatial dependence of human population substructure [32] to estimate ancestral geographic coordinates of an individual (BAPS2 [33], GENELAND [34], sPCA [35], SPA [36], LOCO-LD [37], TESS [38], or GPS [39] among others).

There are several ways to estimate the unknown number (*K*) of ancestral populations from the data (for example, [40]). In model-based methods, the algorithm is explicitly run by the user at different *K*s. The most supported number of clusters or ancestral components is then ascertained by taking the one that optimizes the parameter of performance of the algorithm (for example, it maximizes the log-likelihood of the posterior in the case of STRUCTURE; minimization of cross-validation error is applied in ADMIXTURE among others). In the case of model-free methods, using their output, a classifier algorithm can be applied in order to identify the number of genetically homogeneous population clusters (see for example [41,42], or [43]). One exception is sNMF [25], a new algorithm for inferring ancestry proportions. sNMF models the probability of the observed genotypes *p*_*il*_ in individual *i* at locus *l* as a fraction *q*_*ik*_ of *K* ancestral genotype probability *g*_*kl*_, similar in spirit as STRUCTURE or ADMIXTURE:

$$ {p}_{il}(j)={\displaystyle \sum_{k=1}^K}{q}_{ik}{g}_{kl}(j) $$

where *j =* 0,1,2 denotes the number of alleles. However, this algorithm does not make any assumption about HWE in the ancestral populations. The corresponding matrix representation is *P = QG*, where the unknown *Q* and *G* can be estimated by nonlinear matrix factorization. This is achieved by means of minimizing two least square criteria:

$$ L{s}_1=\left|X-QG\right|\mathrm{and}\kern0.24em L{s}_2=\left|\left({G}^T;\sqrt{\upalpha}\kern0.24em {1}_K\right){Q}^T-\left({X}^T;{0}_n\right)\right|, $$

where alpha is a regularization parameter, and 1_*k*_ and 0_*n*_ describe a column vector with ones and zeros of size *K* and *n* (see [25] for further details; the semicolon indicates a line break). Starting from random matrices as initial condition, the algorithm applies both criteria consecutively to obtain estimates about *Q* from *Ls*_1_ and *G* from *Ls*_2_, respectively, until convergence has been reached.

Since model-based methods explore the space of possible solutions starting from an initial point, it is recommended to run the algorithm several times at different initial starting points for each proposed *K* and to check for reproducibility of results [44]. Different strategies have been proposed for combining the results from different runs. One possibility is to compute a consensus ancestry value by merging all the solutions [44]. Another is just to take the run that provides the best value of model performance [24].

Usually, investigators apply both model-free (for example, PCA or MDS) and model-based methods (for example, ADMIXTURE, FRAPPE, or STRUCTURE) to the same dataset [45,46]. Plots (and further interpretation) tend to include the solutions of the optimal/best supported number of clusters.

Further improvements on genotyping technology, with the description of millions of single nucleotide polymorphisms (SNPs) in the human genome [15], have allowed the third generation of ancestry methods by modeling the genetic ancestry of local fragments of the genome, such as HapMix or StepPCO scripts [14,47] among others.

### Ups and downs of individual genetic ancestry estimation

Individual ancestry methods can depict a detailed picture of the genetic landscape of human populations [15]. Furthermore, these algorithms are routinely applied to any dataset before conducting a genome-wide association study (GWAS), in order to correct for the putative presence of hidden population substructure [48]. Moreover, they have been used to test the hypothesis of the ancestry origin of the perpetrator at a crime scene in forensic cases [49].

In principle, averaging the fragments of local ancestry over the genome of one individual computes the global ancestry estimation in that individual; similarly, averaging all of the global individual ancestries in one population provides a migration/admixture estimation in that population. Moreover, the mean and variance in the length of the ancestry fragments and the global ancestry proportions can be used to estimate parameters such as the time or migration rate of the admixture event in particular demographic scenarios [50]. Nevertheless, population-based methods are sometimes preferred over global or local ancestry methods [18,51]. The main reason is that the results of global and local ancestry methods can be particularly difficult to interpret [21,52]. For example, several demographic scenarios can produce the same observed admixture pattern in PCA [30,53,54]. In humans, multiple demographic events can be identified in the same geographic area [55]; therefore, it is likely to find an *ad hoc* plausible explanation for any estimated admixture pattern (for example, see [53]). The presence of unequal sample size of the (*a priori* unknown) populations can also bias the output of some algorithms, such as PCA [30,56]; the presence of highly genetically related individuals and genetic outliers can also bias the output from different algorithms (such as in the case of PCA, [57]). Furthermore, the outcome from the different algorithms can differ substantially even for the same dataset [58]. Ultimately, there is the question of what a proposed ‘ancestral population’ is. By definition, since new populations appear by splitting from previous ones, population ancestry (and hence genetic admixture) can be defined at different time scales, taking into account that all individuals from a species ultimately share a common ancestral origin. However, this population ‘birth and death’ process is not really modeled in the model-based methods (and by default, neither is it in the model-free methods); in contrast, it is one of the main goals of population-based methods, conditioned to the proper definition of ‘what a current population is’.

We exemplify some of these caveats using unsupervised analyses from four ascertained global-based algorithms on simulated and real data using the default parameter settings from each algorithm. In particular, we consider ADMIXTURE [24], sNMF [25], fastSTRUCTURE [59], PCA [27], and MDS in PLINK [28]. This selection is based on methodological, historical, and computational characteristics. For example, we did not consider fineSTRUCTURE [60], a recently developed algorithm with enhanced power for detecting population substructure [61], because of its computational burden when the number of SNPs and sampled individuals are large (see the manual of fineSTRUCTURE and chromoPainter for details). The first two methods represent model-based algorithms. ADMIXTURE [24] is a maximum likelihood algorithm. It can be considered the gold standard of model-based methods; it is relatively fast and allows for the use of a large number of SNPs and samples. fastSTRUCTURE is a new software that implements a Bayesian framework similar to STRUCTURE [22]. However, in contrast to STRUCTURE, fastSTRUCTURE allows the fast analysis of a large number of samples and SNPs. PCA, MDS, and sNFM are model-free methods. PCA and MDS are based on eigenvalue decomposition. They produce almost identical results in real data [62,63]; therefore, we have used either one or the other indistinctly in the different simulations. sNMF [25] is a novel software which in principle produces very similar results to ADMIXTURE [24] but at a computationally faster speed.

**Figure 1:**
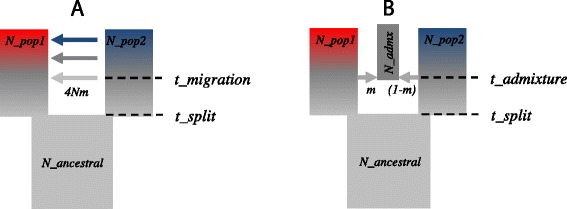
**Basic admixture models commonly used in population genetics.** Each rectangle represents a population. Both models consider one initial ancestral population (gray color) that splits into two new populations *t_split* generations ago. Each of the new populations evolves without exchanging migrants for a period of time, during which genetic differentiation between them can take place as exemplified by the presence of a different color. **(A)** Continuous gene flow (CGF) model. The blue population contributes 4 *Nm* chromosome migrants to the red population from time point *t_split* onwards, replacing the same number of chromosomes from this population. **(B)** Hybrid (HI) model. At *t_admixture*, there is a single event of admixture, and a new hybrid population is created from *m* fraction of chromosome migrants from the blue population and 1*-m* fraction of migrants from the red population. After this event, each population continues to evolve independently. Adapted from [20].

We focused our analyses on two simple, controlled, demographic models. The first demographic model describes an ancestral population that splits *t* generations ago in two populations. In one version of the model, the two descendent populations start evolving independently. In another version, migration between the two populations is allowed. The second model comprises an ancestral population that splits in two, which after a certain number of generations evolving with a genetic barrier, create a new population by admixture (see Figure 1). Because of their simplicity, the proposed demographic models fit better into the assumptions of model-based methods. Furthermore, it has been shown that the first dimension of the PCA can differentiate the genetic ancestry of populations, and it is indicative of the ancestry proportions in the admixed populations [30]. In our analyses, we used markers in linkage equilibrium; this condition was either imposed on the simulator (case of ms simulations) or achieved by the use of commonly applied LD pruning techniques. Therefore, any difference observed in the estimated ancestry proportions must reflect inner algorithmic assumptions or sensitivity to the modification of the considered parameters.

### Performance of global-based algorithms to estimate genetic ancestry on two simulated populations

#### Two populations with a genetic barrier

The results from the two-population model (Figure 1A) with a genetic barrier and the details of the implementation are shown in Tables 2 and 3.

**Table 2:** **Default parameter used in two-population models, with and without migration**

**Parameter**	**Abbreviation**	**Default value**
Sample size population 1	n1	100
Sample size population 2	n2	100
Number of independent SNPs	nsnps	5,000
Mutation rate (length)^a^	theta	2
Effective population size^b^	N1, N2	10,000
Divergence time	T1	2,000
Constant migration rate	4 *Nm*	0

^a^The scaled mutation rate theta = 2*Ne*mu = 2 describes a region of about 2 kb assuming a mutation rate of 2.5e − 8. ^b^The effective population size corresponds broadly to that of Africa.

**Table 3:** **Results from the two-population model simulations**

**Variable**	**sNMF (R2)**	**Admixture (R2)**	**fastStructure (R2)**
Sampling depth, n1, n2			
8	99.92	100	39.56
10	99.83	100	34.03
20	99.87	100	100
40	99.81	100	100
100	99.74	100	100
Uneven sampling, n1			
8	98.94	99.45	98.59
10	99.43	99.78	99.32
20	99.61	100	92.21
40	99.67	100	100
100	99.74	100	100
Sequencing depth, nsnps			
10	3.13	0.65	18.51
50	66.56	75.54	74.42
100	85.33	92.95	91.89
500	96.78	99.87	99.93
1,000	98.62	99.99	100
5,000	99.74	100	100
Population size, theta			
1	99.73	100	100
2	99.74	100	100
5	99.74	100	100
10	99.72	100	100
Effective population size, N2			
100	99.98	100	100
2,500	99.94	100	100
7,500	99.82	100	100
10,000	99.74	100	100
Divergence time (*F*_*st*_), *T*/(4 N_1_)			
0.000075	0.54	0.38	0.01
0.00025	0.24	0.03	0
0.00125	6.19	0.03	0.24
0.0025	69.36	95.28	0.53
0.0125	98.36	100	100
0.05	99.74	100	100
Constant migration rate, 4 *Nm*			
0.1	99.77	100	100
1	99.78	100	100
5	99.56	100	100
10	99.15	99.99	100
50	93.95	99.98	33.3
100	41.61	94.06	0.56

We simulated two populations using ms [75], which splitted and evolved independently *t* generations ago. See Table 1 for default parameters. Each simulation comprises 1,000 independent regions of 2 kb, from which one SNP per region is sampled at random. Each parameter set was replicated ten times. For each algorithm, the estimated ancestry proportions over the different runs were sorted according to the expected ancestry matrix denoting the true population labels using CLUMPP [44]. From this, standard denoted demographic parameters were successively varied to exemplify the impact on the estimates. We report the coefficient of determination that can be understood as the percentage of the true outcome.

**Figure 2:**
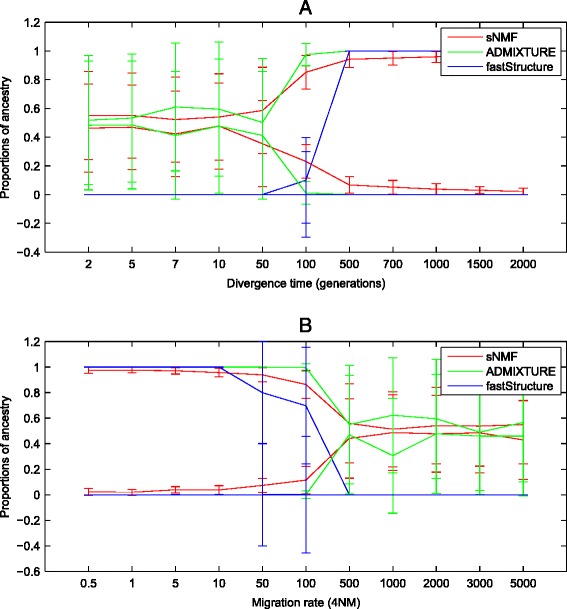
**Estimated proportions of ancestry from the continuous gene flow (CGF) model (see main text).** See Table 2 for default parameters. **(A)** Results for varying divergence time while keeping the migration rate constant at 4 *Nm* = 50. **(B)** The estimated ancestry proportions for keeping the divergence time constant at *T* = 10 while varying the migration rate. Error bars denote the standard deviation of the estimated ancestry proportion per population. Simulations were produced using the following ms command [75]: ms 200 5000 -t 2 -I 2 100 100 -em 1 2 2000 -n 2 1 -ej 2 1.

Overall, sNMF and ADMIXTURE show similar results and outperform fastSTRUCTURE for most of the considered demographic values (see Table 4). Nevertheless, the predictive power of ADMIXTURE is slightly higher than that of sNMF (100% compared to 99% in most cases). Low sample size decreases the power mostly in fastSTRUCTURE (for *n* = 8, fastSTRUCTURE: 35%, sNMF: 99%, ADMIXTURE: 100%), whereas uneven sampling does not influence the estimates of the ancestry components with any of the programs. The number of SNPs has a strong impact on all programs. When only very few sites are available (that is, less than 50 snps), fastSTRUCTURE produces the best outcome. This is not surprising, as ADMIXTURE and sNMF have been particularly developed to consider a dense number of markers [25]. The effective population size and differences in population size did not show any direct impact on the results, which however might matter in combination with divergence time. The power for all programs decreases dramatically for populations that do not exhibit substantial population subdivision due to low divergence times or high migration rates, mostly for fastSTRUCTURE. Reliable ancestry estimates are possible for *t* > 0.0125 that correspond to *F*_*st*_ *>* 0.0124 [64]. The counter effect of constant migration becomes evident for a migration rate of 4 *Nm >* 10 (see Figure 2B), which homogenizes the population. Sampling more sites is likely to increase the sensitivity to detect both effects.

**Table 4:** **Results from admixture simulation with changing parameter in the HI model from HapMap III data**

**Parameter**	**sNMF (R2)**	**ADMIXTURE (R2)**	**fastSTRUCTURE (R2)**
Sample size			
8	98.2	99	86.66
10	99.5	99.52	98.69
20	99.74	99.82	99.71
40	99.85	99.9	99.86
50	99.87	99.93	99.9
100	99.91	99.95	99.95
nsnps			
5	4.56	15.38	19.44
10	15.92	47.37	46.2
50	80.62	86.31	86.89
100	89.67	93.04	93.33
500	98.46	99.07	99.11
1,000	99.19	99.54	99.56
5,000	99.84	99.92	99.91
10,000	99.91	99.95	99.95
Nbreaks			
5	88.82	88.37	87.46
10	94.38	94.86	94.43
50	98.74	98.87	98.8
100	99.33	99.41	99.38
500	99.81	99.85	99.84
1,000	99.86	99.91	99.9
5,000	99.91	99.94	99.94
10,000	99.91	99.95	99.95
alpha			
0.01	99.94	99.99	99.99
0.03	99.93	99.97	99.95
0.07	99.93	99.97	99.92
0.1	99.93	99.97	99.91
0.3	99.91	99.96	99.95
0.5	99.91	99.95	99.95

The admixed population was generated from the African (YRI) and European (CEU) population from HapMap III. A sample from an admixed population is known to consist of a mosaic of chromosomal regions or blocks from the ancestral population. With increasing time since the admixture event, these regions are becoming broken up into smaller pieces through recombination that is denoted by the number of break points (Nbreaks). Individuals from the synthetically admixed population were sampled randomly from blocks from source populations, respectively (the defined admixture proportions, alpha). Finally, a subsample (nsnps) of uniformly distributed sites was chosen. The distance of the sites has been chosen to be greater than 1 Mb to assure linkage equilibrium.

#### Migration between the two descendent populations (continuous gene flow model)

In addition, we studied the parameter range where migration becomes detectable depending on the start time and rate of migration in the continuous gene flow (CGF) model (see Figure 1A for the model and Figure 2 for results). Keeping the migration rate fixed at high migration rate (4 *Nm =* 2,000), the populations become distinguishable if the migration starts before 100 generations backward in time (Figure 2B). Beyond that value, the effect of migration is so strong that the two populations appear to be panmictic. In contrast, when fixing the start time of migration at ten generations, we observe that all populations become recognizable by all programs for 4 *Nm <* 500. The estimated proportions of ancestry do not match the proportion of migrants over time. A possible reason is that there is a continuous gene flow from one population into the other so that recombination has not enough time to produce the homogeneous mosaic of ancestral fragments that is emerging from the HI model (see below). Therefore, the migration rate cannot be inferred from this analysis.

**Figure 3:**
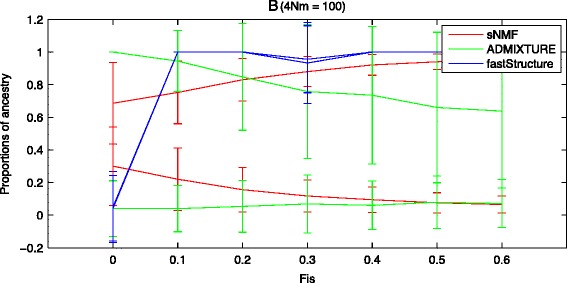
**Migration and inbreeding using the two-population model (see legend of Figure** **2** **for ms command).** Inbreeding was simulated by a reduction of the heterozygote genotypes proportional to the given *F*_*is*_ value (see main text for details).

We further investigated how the presence of hidden inbreeding affects the estimated genetic ancestry proportions from each algorithm. We used the two-population model with constant migration (4 *Nm =* 100) as previously described. In each simulation, a fraction of heterozygote genotypes was decreased proportional to the *F*_*is*_ (for example, [65]) by replacing them by random homozygote genotypes in one population. We estimated the genetic ancestry by the different programs (see Figure 3 for results). The migration has a homogenizing effect on the genetic variation in both populations, whereas the inbreeding in one of the populations results in the opposite pattern. For low *F*_*is*_ values (*F*_*is*_ < 0.1), we observe that sNMF and fastSTRUCTURE indicate correctly the effect of migration in their estimates (see Figure 3). In contrast, for high *F*_*is*_ values (*F*_*is*_ > 0.1), the genetic variation is more divergent in sNMF and fastSTRUCTURE; in contrast, both populations appear more similar with ADMIXTURE. Therefore, sNMF and fastSTRUCTURE seem to provide better ancestry estimates compared to ADMIXTURE, particularly when inbreeding is high (*F*_*is*_ > 0.1). If migration is absent, inbreeding has a minor effect on the ancestry estimates from the different algorithms (data not shown).

**Figure 4:**
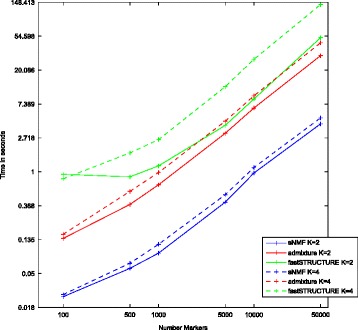
**Netto time estimates for fastSTRUCTURE, sNMF, and ADMIXTURE.** Mean time estimates of the termination of the respective programs from ten independent replications. We simulated 100 chromosomes from two populations with an effective population size of 10,000 and a Ne*m = 20 using ms [75] (see legend of Figure 2 for command details). The termination time can be expected to scale similarly as the number of used SNPs given the complexity of the programs.

For completeness, we studied the running time performance of each algorithm as a function of the number of considered SNPs and for either *K* = 2 or *K* = 4 assumed ancestral populations (see Figure 4). We observed that sNMF shows the lowest running times for a given number of SNPs and *K*, followed by ADMIXTURE. In contrast, fastSTRUCTURE exhibits the worst runtime and scaling with higher *K* as expected from the complexity described above.

### Performance of the algorithms on the hybrid admixture (HI) model

#### Simulated data

Analyses focused on the estimated individual ancestry proportions in the hybrid population using the HI model (Figure 1B). We compared them with the real proportions of genomic admixture in each individual; this measure was estimated for each simulation by tracing back the ancestry of the genomic fragments that compose the genome of each admixed individual to either of the two parental populations. Therefore, in contrast to other approaches, which produce admixed individuals in forward generations from sampled real populations (that is, African Americans have been modeled as a mixture of CEU and YRI individuals from HapMap III [66]; also see the next section), we avoid the artificial introduction of strong bottlenecks.

**Figure 5:**
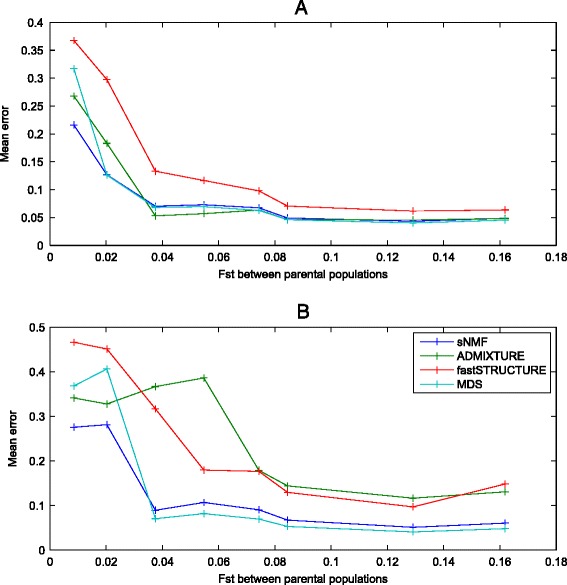
**Estimated error in the estimated individual admixture proportions from the simulated admixed population (HI model).** We used an extended version of the backward demographic simulator described in [76] that includes recombination and different types of mating and allows for ancestry painting [14]. Over all parameters that are defined in this model [19], we varied the time of split of the ancestral populations, which ranged between 50 and 2,000 generations among simulations. Each simulation generated 75 (25 by population) full human genomes with 22 diploid chromosomes (*l*) with the following sizes: 13.65, 13.15, 11.20, 10.65, 10.20, 9.65, 9.35, 8.50, 8.40, 8.95, 7.95, 8.65, 6.35, 5.80, 6.30, 6.75, 6.50, 5.95, 5.40, 5.40, 3.10, and 3.65 Mb [77]. The mutation rate was set to 2.5 × 10^−8^ [78] and the recombination rate to 1.8 × 10^−8^. PLINK was applied to exclude SNPs with minor allele frequency less than 0.05 and LD (default PLINK --indep 50 5 2). The effective population sizes of the parental and hybrid populations were set to 5,000 diploid individuals; the time of admixture was ten generations ago, and each parental population equally contributed to the admixed population. By this way, we minimized the putative effect of genetic drift in the admixture proportions of the hybrid population. Furthermore, in order to include the effects of bias sample size, we repeated all the analyses with 1:1 **(A)** and 1:5 **(B)** parental population size ratios. Four different algorithms were considered: sNMF, ADMIXTURE, fastSTRUCTURE, and MDS. In the case of MDS, ancestry proportions of each individual from the admixed population were estimated as the relative position in the first dimension in relation to the mean estimated coordinate of the parental populations.

As seen in Figure 5, the error of the estimated ancestry proportions differ based on the software, the amount of genetic differentiation present among the parental populations, and the ratios of sampled individuals between the parental populations. With the same number of sampled individuals by parental population, ancestry proportions estimated by fastSTRUCTURE show the largest deviation to the real ancestral proportions in all the simulations. In all cases, admixture proportions in the admixed population tend to be better estimated if the parental populations are genetically differentiated (*F*_*st*_ > 0.1); nevertheless, even in that case, the mean difference between the estimated and the real admixture proportion can reach 5% in the case of sNMF and MDS, and 6% in the case of fastSTRUCTURE. Unequal sample sizes of the parental populations also affect the performance of the different algorithms. ADMIXTURE and fastSTRUCTURE show a systematic error bias in the estimation of the admixture proportions in the hybrid population when there is unequal sample size in the parental populations, independently of the amount of population differentiation among the parental populations.

#### Real data from HapMap III data

Simulations from synthetically generated admixed populations from African (YRI) and European (CEU) as ancestral populations were produced (see Table 4 for results and clarification of the applied methodology). We use the number of breakpoints to mimic the time of admixture [14] and sampled SNPs with a minimum distance of 1 Mb to ensure linkage equilibrium. The results for sample size, number of SNPs, and admixture time, represented here as the number of breaks, are quite similar to the two-population simulations above. The power of sNMF and ADMIXTURE is quite comparable. fastSTRUCTURE loses power more rapidly with lower sample size and maintains a better power for low numbers of SNPs. All programs have an equally high power to estimate the ancestry components.

## Conclusions

Identifying hidden population substructure in the genome of an individual is important for a number of scientific disciplines. So far, the proposed algorithms are invaluable tools for detecting and controlling for the presence of hidden population substructure. In the simplest demographic models, these methods can also be used to estimate demographic parameters. However, interpreting the output of each algorithm from an evolutionary point of view can be difficult. Different demographic scenarios can lead to the same ancestry estimates, and different estimates can be retrieved when applied to the same dataset. Extrapolating the results from our simple simulations to real data (that is, suggesting which is the best algorithm) can be misleading; except for cases such as the admixture of European and Sub-Saharan African populations in the US [67], admixture usually involves more than two parental populations (for example, Latin America, although see [68]). In addition, parental populations tend to show a non-negligible gene flow [61] with admixed populations that can substantially differ in the effective population size compared to the parental populations (for example, see the European Romani [46]), while usually the parental populations are unknown.

The number of SNPs and sample size seem to be a limiting factor in all the algorithms that we have tested; therefore, it would be recommended to use as many markers (conditioned in the absence of LD when required by the algorithm) and samples as possible. However, in our simple model, we observe already good estimates for >10 samples and >1,000 markers. In case fewer markers are available, fastSTRUCTURE provides the best estimates followed by ADMIXTURE and sNMF. Furthermore, it is recommendable to run more than one algorithm on the same data at the same time given the observed diversity of results, different sensitivity to biased sample size of the different algorithms, and ancestry noise. In this sense, combining global ancestry and population ancestry methods (for example, [69]), or using the output from these algorithms as summary statistics [40], can improve the identification of population substructure. Finally, although they can be used to provide hypotheses about the origin and evolution of populations, it is recommended to test the evolutionary hypotheses by means of other methods [46], rather than providing an *ad hoc* interpretation; in particular, any demographic interpretation from these methods should be further validated by means of demographic simulations, showing that the proposed demographic model can produce the observed output of genetic ancestry.

## Abbreviations

AMOVA: Analysis of molecular variance
CGF: Continuous gene flow model
GAMOVA: Generalized analysis of molecular variance
GWAS: Genome-wide association study
HWE: Hardy-Weinberg equilibrium
MDS: Classical multidimensional scaling, also called principal coordinate analysis
PCA: Principal component analysis
SNP: Single nucleotide polymorphism
